# Lycopene Inhibit IMQ-Induced Psoriasis-Like Inflammation by Inhibiting ICAM-1 Production in Mice

**DOI:** 10.3390/polym12071521

**Published:** 2020-07-09

**Authors:** Chun-Ming Shih, Chi-Kun Hsieh, Chien-Yu Huang, Chun-Yao Huang, Kuo-Hsien Wang, Tsorng-Harn Fong, Nguyen Thi Thu Trang, Kuan-Ting Liu, Ai-Wei Lee

**Affiliations:** 1 Department of Internal Medicine, School of Medicine, College of Medicine, Taipei Medical University, Taipei 11031, Taiwan; cmshih53@tmu.edu.tw (C.-M.S.); cyhuang@tmu.edu.tw (C.-Y.H.); 2 Cardiovascular Research Center, Taipei Medical University Hospital, Taipei 11031, Taiwan; 3 Taipei Heart Institute, Taipei Medical University, Taipei 11031, Taiwan; 4 Department of Medical Education and Research, Kaohsiung Veterans General Hospital, No. 386, Dazhong 1st Rd., Zuoying Dist., Kaohsiung City 81362, Taiwan; faceeric0302@yahoo.com.tw; 5 Department of Surgery, School of Medicine, College of Medicine, Taipei Medical University, Taipei 11031, Taiwan; cyh@tmu.edu.tw; 6 Division of General Surgery, Department of Surgery, Shuang Ho Hospital, Taipei Medical University, Taipei 11031, Taiwan; 7 Department of Dermatology, Taipei Medical University Hospital, Taipei 11031, Taiwan; khwang40@gmail.com; 8 Department of Anatomy and Cell Biology, School of Medicine, College of Medicine, Taipei Medical University, Taipei 11031, Taiwan; thfong1023@gmail.com (T.-H.F.) ; jack841023yes@gmail.com (K.-T.L.); 9 International Ph.D. Program for Cell Therapy and Regeneration Medicine, College of Medicine, Taipei Medical University, Taipei 11031, Taiwan; nguyenthutrang0109@gmail.com

**Keywords:** lycopene, psoriasis

## Abstract

Lycopene is the most abundant carotenoid in tomatoes, which has been identified to have the properties of anti-inflammation in addition to the capability to inhibit the expression of adhesion molecules. Intercellular adhesion molecules play a critical role in the pathogenesis of psoriasis. Here, we report that the topical use of a lycopene decreased imiquimod (IMQ)-induced psoriasis-like inflammatory responses, the progress of which was based on adhesion molecules. In vitro analysis showed that lycopene decreased keratinocyte and monocyte adhesion. Evidence suggests that intercellular adhesion molecule-1 (ICAM-1) is a main mediator of psoriasis pathogenesis. Therefore, it will be interesting to investigate the factors that contribute to the lycopene-mediated inhibition of ICAM-1 expression in psoriasis. We expect that lycopene will with potential value in the treatment of psoriasis.

## 1. Introduction

Psoriasis is a chronic inflammatory skin disease that is characterized by the formation of scaly, erythematous plaques. Psoriasis affects approximately 2% of the population [1]. The pathogenesis of psoriasis is complicated. According to a previous report, activated T cells are a major component of the inflammatory infiltrate of psoriatic lesions [2]. Adhesion molecules include intercellular adhesion molecule-1 (ICAM-1) and vascular cell adhesion molecule-1 (VCAM-1), which play an important role in the infiltration of T cells into psoriasis lesion sites [3]. We know that PUVA and UVB therapy are important clinical treatments for psoriasis vulgaris. It has been demonstrated that UVA and UVB therapies reduce the expression of adhesion molecules [4]. In recent years, there has been more evidence that indicates that the interleukin-23 (IL-23) and IL-17A signaling pathway plays a main role in the development of psoriasis [5]. Tasato et al. found that intercellular IL-23p19 increased the expression of adhesion molecules on the surface of endothelial cells [6]. Specific IL-23p19 blockade with high-affinity monoclonal antibodies seems to be able to treat psoriasis [3]. Many therapeutic agents are available for the treatment of psoriasis. Include creams and ointments, phototherapy, and oral or injected medication. Nevertheless, none of these agents are absolutely effective and safe for the treatment of this disease. 

Lycopene is a hydrocarbon phytochemical that belongs to the tetraterpene carotenoid family and is found in red vegetables and fruits, such as watermelon, pink guava, and tomato. Lycopene has been found to be active in many cell lines, including prostate cancer and oral cancer cell lines [7]. Lycopene also inhibits the metastasis of human liver adenocarcinoma *SK*-*Hep*-*1* cells [8]. Lycopene is also a beneficial treatment for people with cardiovascular diseases [9]. Another study showed that increasing lycopene levels in the blood can reduce oxidative stress and improve endothelial cell function [10]. In addition, lycopene also inhibits NF-κB activation and adhesion molecule expression in endothelial cells [11]. In stomatology, lycopene plays a multifunctional role as a nonsurgical support in the treatment of oral diseases, including leukoplakia and oral submucous fibrosis [12]. Therefore, we know that lycopene, which is the most abundant carotenoid in tomato, may enhance human health benefits in many systems. According to a previous dermatology study, lycopene protects human keratinocytes against full spectrum UVR damage [13]. Although the HaCaT cells immortal, the virtually normal degree of morphologic differentiation was further substantiated by the regular spatial distribution of epidermal differentiation products. Furthermore, the pattern of keratin expression, including the suprabasal epidermal keratins, was almost identical to those seen in transplants of normal keratinocytes [14]. According to previous studies HaCat cells widely used as a model of psoriatic dermatitis. Hacat could be a promising candidate for study psoriasis dermatitis. Lycopene may have potential possibility for the prevention and treatment of psoriasis. The results show that imiquimod (IMQ)-induced psoriasis lesions in mice are similar to human psoriasis lesions with regard to not only their histological and phenotypic characteristics but also their development. This study investigated the effects of lycopene gel and lycopene by oral gavage in an IMQ-induced psoriasis-like mouse model. We anticipated obtaining evidence that supports a role for lycopene in treatment of psoriasis.

## 2. Results

### 2.1. Adequate Dosage of Lycopene for Animals Study

**Figure 1:**
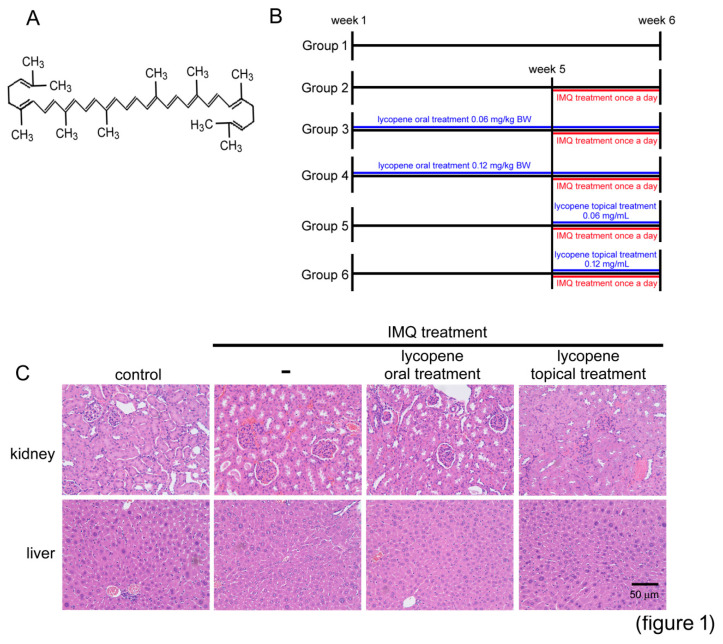
Lycopene treatment did not affect liver and kidney function. (**A**) The molecular structure of lycopene. (**B**) The experimental design and animal groupings are presented. (**C**) The mice were pretreated by oral administration of 0.12 mg/kg BW/day or by topical treatment of 0.12 mg/mL lycopene followed by imiquimod (IMQ) stimulation. Representative photographs of kidney and liver sections stained with hematoxylin and eosin and analyzed using microscopy at 200× magnification.

During the experimental period, there were not significant differences in the final weight and weight gain of the animals (data not shown) among the groups. As shown in Table 1, between the oral and topical lycopene treatment groups, the aspartate aminotransferase (AST), alanine aminotransferase (ALT), creatinine, and blood urea nitrogen (BUN) levels were not significantly different at the end of the study. Morphometric studies of the kidney and liver were performed to measure that the using of lycopene dosage in the animal was not toxic. According to the morphological assay, the topical treatment of 0.12 mg/mL lycopene for 7 days and the oral treatment of 0.12 mg/kg BW/day for 42 days did not induce kidney injury, including the narrowing of the Bowman space, glomerulonephritis, and compression of capillaries, which compared with the control animals. The livers from the lycopene-administrated mice did not show macrocellular fatty or microcellular changes, vascular congestion or periportal fibrosis, and feathery degeneration (Figure 1C). Thus, mice administrated with lycopene via topical (0.12 mg/mL ointment) and oral (0.12 mg/kg BW/day) treatment presented normal liver and kidney functions and histology.

### 2.2. IMQ Induces Psoriasis-Like Phenomena in Mice, and Lycopene Decreases the Severity of Symptom Appearance

**Figure 2:**
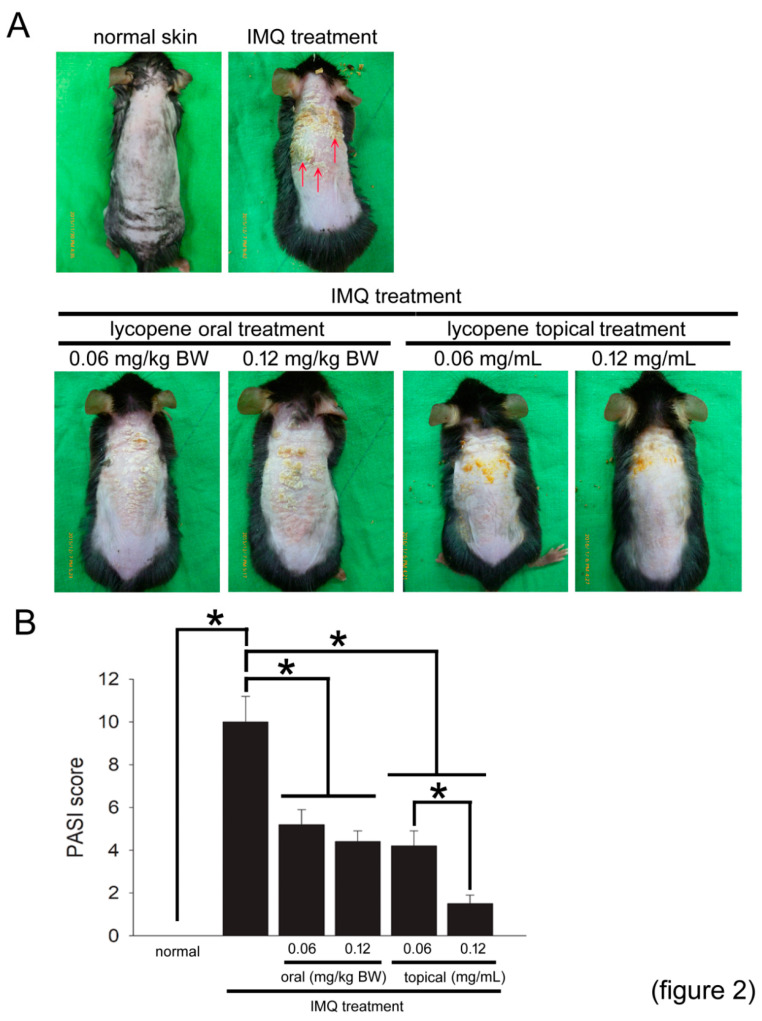
Lycopene improves psoriasis-like phenomena in mice. (**A**) Mice in each group were presented in representative photos. Representative photos of the mice that received IMQ treatment with or without oral administration of and topical treatment with lycopene. The arrows indicate psoriasis-like and scared skin. (**B**) The PASI score is shown as a bar graph. The results are expressed as the mean ± SD. A * *p* < 0.05 was considered statistically significant.

The representative photos (Figure 2A) show psoriasis-like skin in the mice treated with IMQ without or with lycopene administration. IMQ treatment significantly caused the psoriasis-like skin occurrence in mice. Increased psoriasis-like skin lesions and scarring (indicated by the arrowhead) were observed in IMQ-treated mice compared with untreated normal mice. The Psoriasis Area and Severity Index (PASI) score was used to quantify the psoriasis-like skin severity shown as a bar graph. The PASI score was increased in normal mice treated with IMQ. Interestingly, both the oral administration of lycopene (0.06 and 0.12 mg/kg BW/day) and the topical treatment with lycopene (0.06 and 0.12 mg/mL of ointment) efficiently decreased the PASI score in IMQ-treated mice. Additionally, a dosage-dependent effect was observed in the groups treated with topical lycopene at dosages between 0.06 and 0.12 mg/mL. These results suggest that lycopene decreases psoriasis-like phenomena, and topical treatment with an ointment provides a better effect in IMQ-treated mice.

### 2.3. Lycopene Decreases the Serious Epidermis Hyperplasia in IMQ-Treated Mice 

**Figure 3:**
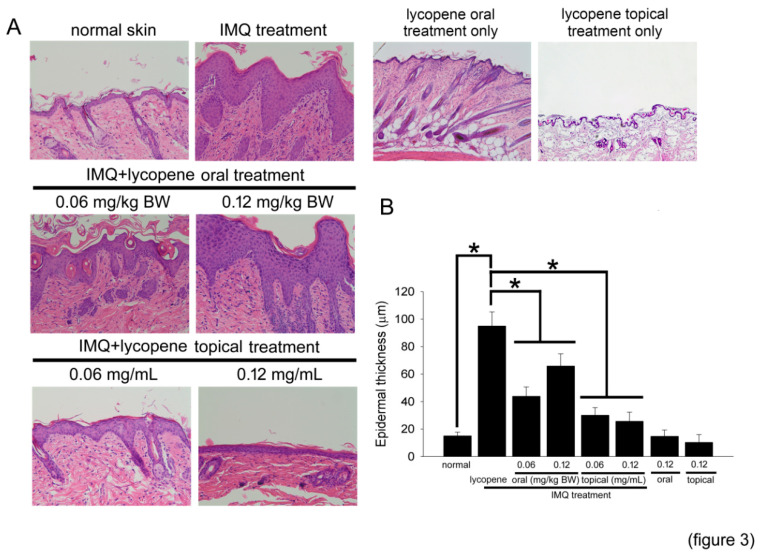
Lycopene inhibits epidermal hyperplasia and proliferation in IMQ-treated mice. (**A**) Structural features characteristic of IMQ-induced psoriasis-like skin were analyzed in H&E stained sections and analyzed using microscopy at 200× magnification. (**B**) The epidermal thickness was quantified and is presented as a bar graph. The results are expressed as the mean ± SD. * *p* < 0.05 was considered statistically significant.

To further study the effects of lycopene on epidermal proliferation, hematoxylin and eosin (HE)-staining were used to analyze the structural features characteristic of animals′ IMQ-induced psoriasis-like skin. In Figure 3A, a thin epidermis was observed in normal skin. IMQ significantly induced hyperplasia of the epidermis. Furthermore, the skin of mice both in 0.06 and 0.12 mg/kg BW lycopene oral treatment for 6 weeks plus IMQ treatment for 1 week exhibited slight keratinocyte hyperplasia. Additionally, topical treatment with lycopene had more obvious effects than oral administration. These results suggest that lycopene may inhibit epidermal hyperplasia and proliferation in IMQ-treated mice.

### 2.4. TNF-α Induces Keratinocyte Activity and Increases Monocytic Cell Adhesion, Which Is Inhibited by Lycopene Treatment

**Figure 4:**
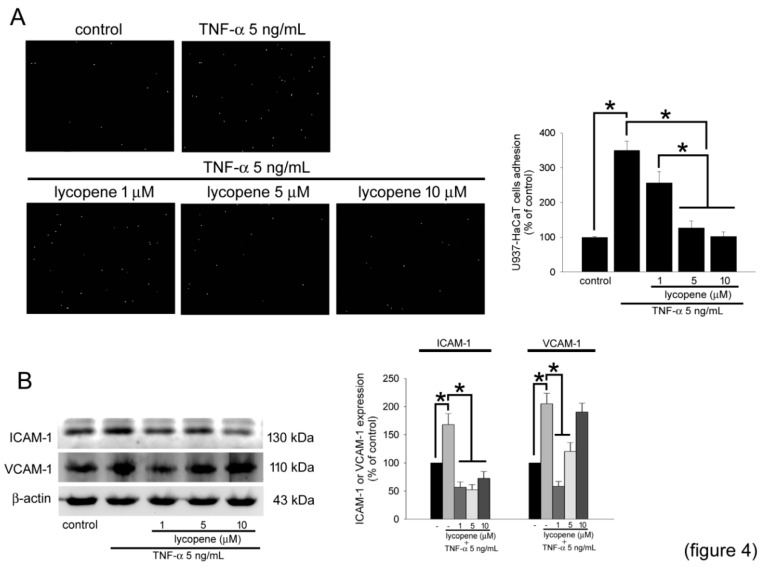
Lycopene inhibits U937 cell adhesion and adhesion molecule expression in TNF-α-stimulated HaCaT cells. (**A**) HaCaT cells were pretreated with 1–5 μM lycopene for 24 h and treated with 5 ng/mL TNF-αα for 4 h. After incubation of BCECF/AM-labeled U937 cells for 1 h, the adhesion of fluorescent U937 cells was observed using fluorescence microscopy. The bar graph shows the quantification of the adherent U937 cells. (**B**) HaCaT cells were treated with 5 ng/mL TNF-α for 4 h with or without preincubation with 1–5 μM lycopene. The Western blot analysis was conducted to analyze the extracted total protein. The loading control was used by β-actin. Densitometer was used to quantify the density of each band. The results are expressed as the mean ± SD. * *p* < 0.05 was considered significant difference, *n* = 3.

TNF-α simultaneously increased the adhesion of keratinocytes and monocytes, which plays a key role in psoriasis. Therefore, we performed an adhesion assay to study the effect of lycopene on TNF-α-stimulated keratinocyte-monocyte adhesion. Figure 4A demonstrates that compared to cells in the control group, the U937 cells in the TNF-α stimulation group exhibited greater adhesion. In the cells stimulated with TNF-α after pretreatment with lycopene, the fluorescence of 2,7-bis(2-carboxyethyl)-5(6)-carboxyfluorescein acetoxymethyl ester (BCECF/AM)-labeled U937 cells in the culture dish was significantly decreased, especially in the groups treated with 5 and 10 μM lycopene. Western blotting was performed to analyze the roles of the adhesion molecules ICAM-1 and VCAM-1 in HaCaT cells. In Figure 4B, TNF-α may significantly induce the expression of ICAM-1 and VCAM-1 in HaCaT cells. Interestingly, pretreatment with 1–5 μM lycopene may decrease ICAM-1 expression under conditions of TNF-α stimulation. In contrast, treatment with only 1 μM lycopene may inhibit the increase in VCAM-1 in TNF-α-stimulated HaCaT cells. These results suggest that lycopene may inhibit HaCaT-U937 cell adhesion, which may be mediated by the inhibition of ICAM-1 and VCAM-1.

## 3. Discussion 

Nutraceuticals are natural, nutritional compounds that can be efficacious in preventative medicine or in the treatment of disease. Several foods, such as fruits, vegetables, cereals, and legumes [15] supplements, have been shown to protect against the development of disease. In a previous study, we have shown that the current medical treatments for chronic diseases are insufficient for some particularly high-risk patients [16]. For example, dyslipidemia is a chronic disease that increases the risk of CVD and psoriasis [17]. Dyslipidemia is the elevation in plasma cholesterol and triglycerides that contributes to the development of atherosclerosis [18]. Experimental studies have indicated that grape polyphenols may affect plasma lipid concentrations [19]. Therefore, nutraceuticals may have potential benefits in the treatment or prevention of some chronic diseases, including psoriasis. Lycopene is a nutraceutical and is the primary carotenoid in human plasma, it is a natural pigment synthesized by plants and microorganisms. Red fruits and vegetables, including watermelons, tomatoes, pink grapefruits and pink guavas, contain lycopene. Processed tomato products, such as juice, paste and soup, are good dietary sources of lycopene [20].

Atopic dermatitis is a long-term type of inflammation of the skin. It results in itchy, red, swollen, and cracked skin. Oral administration of lycopene prevents atopic dermatitis in hairless mice [21]. Another study showed that increasing the lycopene levels in the blood can reduce oxidative stress and improve the function of endothelial cells [10]. However, the effects of lycopene and tomato sauce is still unknown. Until 2016, Lorelei et al. indicated that lycopene and tomato sauce showed similar effects on prostate cancer tumors. However, the mechanisms exhibited by lycopene and tomato sauce were different. However, the lycopene levels in plasma after the consumption of lycopene and tomato sauce were similar [22]. In our study, we used nutraceuticals of lycopene both orally and topically to inhibit epidermal hyperplasia and proliferation in IMQ-treated mice. The results suggest that lycopene has potential benefits in the treatment or prevention of psoriasis.

Adhesion molecules include intercellular adhesion molecules (ICAMs) and vascular cell adhesion molecule-1 (VCAM-1) and have a key role in the inflammatory response [23]. The formation of atherosclerotic plaque and monocyte adhesion to the endothelium is an initial stage of atherosclerosis development [24]. In addition, a dermatology study demonstrated that psoriatic skin widely expressed ICAM-1 and VCAM-1 [25]. A previous study demonstrated that lycopene inhibits ROS production in vitro and prevents LDL oxidation [26]. In conclusion, it was demonstrated that lycopene inhibit IMQ-induced psoriasis-like inflammation in keratinocytes in vitro and in mice. Furthermore, in an IMQ induced psoriasis-like dermatitis mouse model, topical lycopene treatment not only provided local symptomatic benefit but also contributed to the decreases monocytic cell adhesion. Taken together, lycopene, a naturally derived compound, will be of interest for the development of a new drug for psoriasis.

## 4. Materials and Methods 

### 4.1. Drugs and Reagents

IMQ cream (5% *w/w*) was purchased from INOVA Pharmaceutical Co. (Sydney, Australia). Lycopene was purchased from Rexall Sundown, Inc. (Bohemia, NY, USA) for animal studies and purchased from Sigma-Aldrich Co. (Cat. No.: L9879, St. Louis, MO, USA) for in vitro studies. The molecular structure of lycopene is shown in Figure 1A. 

### 4.2. Preparation of Lycopene Gel

The composition of the gel is as follows: lycopen 20 mg, glycerol 1416.7 µL. lycopen was added to the glycerol while stirring. The solution was incubated at ambient temperature and the resulting mixture was stirred continuously until the gel formed. This gel formulation of lycopen showed excellent transdermal effects in an in vitro permeation experiment.

### 4.3. In Vivo Animal Study

#### 4.3.1. Ethics Statement

All the protocols approved by the Institutional Animal Care Committee, Taipei Medical University (No: LAC-2016-0041). The study procedures conformed to the “Guide for the Care and Use of Laboratory Animals” (NIH Publication of USA No. 85-23, revised 1996). 

#### 4.3.2. Animal Study Protocols and Grouping 

Animals were fed a normal murine chow diet (Scientific Diet Services, Essex, UK) and housed in microisolator cages on a 12-h day/night cycle with water ad libitum. Thirty 6- to 8-week-old male C57BL/6 mice (Jackson Laboratory, ME, USA) were used. The psoriasis-like skin inflammation model was established according to a previous reference with modification [27]. Mice were treated with daily topical application of 5% IMQ cream at a dose of 62.5 mg/cm^2^ on the shaved back for 7 consecutive days. All the animals were randomly divided into six groups (*n* = 5; Figure 1B). Group 1 (naïve control): mice were fed a normal chow diet; Group 2: the mice received IMQ administration at the 5th week of beginning of experiment; Group 3: the mice received oral treatment of 0.06 mg/kg body weight (BW) lycopene once a day at the 1st week of beginning of experiment and received IMQ treatment at the 5th week of beginning of experiment; Group 4: the mice received oral administration of 0.12 mg/kg body weight (BW) lycopene once a day at the 1st week of beginning of experiment and received IMQ treatment at the 5th week of beginning of experiment; Group 5: the mice received topical treatment of 0.06 mg/mL lycopene once a day and received IMQ treatment at the 5th week of beginning of experiment; Group 6: the mice in received topical treatment of 0.12 mg/mL lycopene and received IMQ treatment at the 5th week of beginning of experiment. At the end of the experiment (end of 5th week/35th day), the mice were sacrificed, and the treated skin was removed. To evaluate the severity of the inflammation of the shaved skin, the Psoriasis Area and Severity Index (PASI) method was used. Each of four symptoms (erythema, scaling, thickness, and cumulative scores) was scored separately according to the following scale: 0 (not present), 1 (mild), 2 (moderate), or 3 (severe). The scores were summed, and an integration bar was drawn for each group.

#### 4.3.3. Measurement of Biochemical Characteristics

Blood were collected from animals for biochemical assays before entering the experiment, at the end of 6th week, and at sacrifice. The blood were extracted from the mandibular artery into sodium citrate-containing tubes. Plasma AST, ALT, BUN, TG, and total cholesterol were analyzed using the automatic chemistry system (SPOTCHEMTM, SP-4410; Arkray, Japan).

#### 4.3.4. Hematoxylin and Eosin Staining

The animals were sacrificed at the end of 5th week. The back skin was collected and rinsed with ice-cold phosphate buffered saline; fixed by 4% paraformaldehyde, and embedded in paraffin. Then, 5-µm cross-sections were stained with H&E staining. Slides were observed via the TissueGnostics TissueFAXS and HistoFAXS System (TissueGnostics, Vienna, Austria). 

### 4.4. In Vitro Study

#### 4.4.1. Cell Culture

The HaCaT cell was cultured in Dulbecco′s modified Eagle′s medium supplemented with antibiotics (100 U/m penicillin A and 100 U/mL streptomycin), fetal bovine serum (10%), and glutamine (2 mM). Cells were kept in a 37 °C humidified and 5% CO_2_ contained incubator. When performing the experiments, the HaCaT cells were grown to 90% confluence.

#### 4.4.2. HaCaT/THP-1 Cell Adhesion Assay

The HaCaT cells (5 × 10^5^) were seeded into 24-well plates before the assay. Then, the growth medium was supplemented with TNF-α at 5 ng/mL for 24 h. THP1 cells were labeled with 10 μM of 2,7-bis(2-carboxyethyl)-5(6)-carboxyfluorescein acetoxymethyl ester (BCECF/AM, Boehringer-Mannheim) for 1 h at 37 °C in serum-free RPMI 1640 medium; the cells were then washed with PBS to remove the free dye and resuspended in RPMI 1640 containing 2% FBS. One million labeled THP-1 cells were added to each HCAEC-containing well, and the incubation continued for 1 h. The non-adherent cells were removed by three gentle washes with HBSS. The number of THP-1 cells adhered to the HaCaT cells was observed using inverted fluorescence microscopy and counted using a Multilabel Counter Victor^2^ (Wallace, CA, USA) at an emission wavelength of 530 nm and an absorption wavelength of 435 nm after the cells were lysed with DMSO.

#### 4.4.3. Western Blotting Analysis

The total proteins were extracted from keratinocytes. The proteins were separated by SDS-PAGE and transferred to a PVDF membrane. The membranes were hybrid using the anti-VCAM-1 (Santa Cruz, CA, USA) and anti-ICAM-1 (Santa Cruz, CA, USA) antibodies. The loading control was used as an anti-β-actin (Labvision/NeoMarkers, CA, USA) antibody. The proteins were visualized using an enhanced chemiluminescence detection kit (Amersham Biosciences, NJ, USA).

### 4.5. Statistical Analysis

The results are expressed as the mean ± SEM. ANOVA followed by Dunnett′s test was used to analyze the data. *p* < 0.05 was considered as statistically significant. 

## Figures and Tables

**Table 1:** Plasma biochemical characteristics in experimental mice (*n* = 5).

		Time Point	Kidney Function		Liver Function	
			BUN (mg/dL)	Creatinine (mg/dL)	ALT (IU/L)	AST (IU/L)
**naïve control**		Star of week 1	24.6 ± 3.2	0.42 ± 0.04	34.2 ± 3.2	25.3 ± 4.6
		End of week 6	27.4 ± 2.5	0.34 ± 0.05	36.3 ± 2.7	24.7 ± 3.5
**IMQ treatment**	**Non-lycopene treatment**	Star of week 1	26.7 ± 2.2	0.53 ± 0.04	43.4 ± 4.7	26.3 ± 8.2
		End of week 6	25.5 ± 3.5	0.43 ± 0.05	42.2 ± 4.3	31.5 ± 8.7
	**Lycopene oral treatment 0.12 mg/kg BW**	Star of week 1	23.3 ± 3.6	0.54 ± 0.03	42.4 ± 3.8	26.4 ± 7.2
		End of week 6	25.4 ± 2.8	0.46 ± 0.03	43.2 ± 3.5	29.5 ± 4.3
	**Lycopene topical treatment 0.12 mg/mL**	Star of week 1	25.4 ± 4.6	0.52 ± 0.04	41.6 ± 4.5	30.4 ± 6.2
		End of week 6	26.5 ± 3.2	0.44 ± 0.03	36.7 ± 4.2	28.3 ± 3.4

BW—body weight; BUN—blood urea nitrogen; ALT—alanine aminotransferase; AST—aspartate aminotransferase; Values are mean ± SD.
